# What Do We Know About the Genetic Basis of Seed Desiccation Tolerance and Longevity?

**DOI:** 10.3390/ijms21103612

**Published:** 2020-05-20

**Authors:** Hanna Kijak, Ewelina Ratajczak

**Affiliations:** Institute of Dendrology, Polish Academy of Sciences, 62-035 Kórnik, Poland; eratajcz@man.poznan.pl

**Keywords:** seed desiccation tolerance, seed longevity, gene expression, redox genes

## Abstract

Long-term seed storage is important for protecting both economic interests and biodiversity. The extraordinary properties of seeds allow us to store them in the right conditions for years. However, not all types of seeds are resilient, and some do not tolerate extreme desiccation or low temperature. Seeds can be divided into three categories: (1) orthodox seeds, which tolerate water losses of up to 7% of their water content and can be stored at low temperature; (2) recalcitrant seeds, which require a humidity of 27%; and (3) intermediate seeds, which lose their viability relatively quickly compared to orthodox seeds. In this article, we discuss the genetic bases for desiccation tolerance and longevity in seeds and the differences in gene expression profiles between the mentioned types of seeds.

## 1. Introduction

The acceleration of climate change poses new challenges for humanity in the fields of agriculture, forestry and environmental protection. More frequent and longer periods of drought, rising temperatures, and weather anomalies may cause, among other things, a decrease in the quality of harvests, problems with forest stand renewal and migration or even extinction of species. Therefore, it is important to preserve genetic resources, for example, by long-term seed storage in controlled conditions. However, deterioration of seeds is one of the biggest problems affecting gene banks. A gradual decrease in the viability of dry-stored seeds is caused by the aging processes and is revealed by signals such as delayed germination, poor seedling establishment or even a total lack of germination, which may result in reduced crop yields [1,2,3]. Thus, proper seed storage is crucial not only from an economic and social point of view but also for environmental protection [4,5]. The condition of stored seeds depends on many factors, including the storage temperature, moisture content (MC), storage duration, and oxygen pressure [6], but most importantly, on the degree of desiccation tolerance (DT). In general, seeds are divided into three categories: (1) orthodox seeds, which tolerate drying to a moisture content below 7% and storage at −10 °C for a long time [7,8]; (2) recalcitrant seeds, which are very sensitive to desiccation and freezing, as they very quickly lose viability when stored using conventional methods, remaining metabolically active throughout their pre- and post-harvest development [9]; and (3) intermediate seeds, which lose viability relatively quickly compared to orthodox seeds [10]. The vast majority of spermatophytes produce desiccation tolerant (orthodox) seeds, which have an extraordinary ability to survive in extreme environmental conditions [11,12]. Orthodox seeds can be stored for a very long time under gene bank conditions (i.e., temperature below 0 °C, circa 15% relative humidity air) without losing quality [13,14,15]. On the other hand, it is estimated that 8% of flowering plants worldwide display seed desiccation sensitivity (DS, recalcitrance); this percentage increases up to 50% in the case of tropical evergreen rainforests [8,16]. Therefore, it is necessary to understand the molecular mechanisms of both DT and DS for successful long-term seed preservation and biodiversity protection.

DT can be considered when the moisture content of the cytoplasm drops below 10% on a fresh weight basis (or 0.1 g H_2_O/g dry weight) without accumulation of lethal damage [17]. Moreover, DT includes maintaining the ability to successfully rehydrate [18]. This remarkable mechanism was crucial during the colonization of land by the first terrestrial plants [19,20] and allows orthodox seeds in the dry state to remain viable for long periods of time [21]. DT in angiosperms occurs during the late seed maturation stage and requires a complex regulatory network, which is determined by a huge repertoire of genes involved in numerous defense mechanisms [12,21,22,23,24,25].

The acquisition of DT is associated with modification of some intracellular physical characteristics, e.g., deposition of insoluble proteins within vacuoles, to improve mechanical resilience against cell collapse. Starch and lipid accumulation also increase the volume buffering capacity. Additionally, chromatin condensation as well as inhibition of replication and transcription and gradual dismantling of the cytoskeleton accompany DT [26].

The molecular and genetic networks that regulate the DT process remain largely unknown. However, significant achievements have recently been made toward understanding the genetic mechanisms of seed aging and longevity. In this review, we discuss the latest scientific reports on the genetic basis of DT in seeds. A better understanding of genetic DT mechanisms may be useful for both agriculture and environmental protection. Moreover, it may lead to the development of better methods for the preservation of desiccation-sensitive seeds.

## 2. Metabolic Shut-Down

An important factor that allows cells to survive under water deficit conditions is a reduction in metabolic activity and respiratory processes. High metabolic activity has been linked to DS, while a decrease in metabolism is characteristic of DT [15,27,28]. One of the most widespread strategies to slow cell metabolism is to limit the ability of molecules to move around the cytoplasm. To achieve that effect, cells transform the cytoplasm into a glassy state by accumulating the oligosaccharides stachyose and raffinose (RFOs) [11,29,30]. Increases in RFOs and a decreased ratio of sucrose to some RFOs were observed in *Fagus sylvatica* L. seeds with DT [31].

In dry biological tissues, glasses retain the activity of enzymes and conformation of proteins and play a role in the long-term storage stability of seeds [32]. The replacement of water with the abovementioned oligosaccharides during desiccation also maintains the hydrogen bonds required for membrane and protein stabilization [11,21].

As reviewed by Gechev et al. [33], RFOs are also storage carbohydrates, which are mobilized during desiccation for energy and sucrose synthesis. The accumulation of sugars is characteristic of highly desiccation tolerant resurrection plants [34,35,36,37,38], and recent studies showed that orthodox seeds seem to share this defense mechanism. Transcriptomic studies on the most popular plant model organism, *Arabidopsis thaliana*, showed that transcript levels of genes encoding key enzymes in the raffinose pathway, including sucrose synthases, UDP-D-galactose-4-epimerases, galactinol synthases and stachyose synthase, were strongly downregulated in seeds of desiccation-sensitive mutants in comparison with those of the wild type (WT) [21]. On the other hand, upregulation of invertase genes was observed, suggesting that D-glucose and D-fructose were not metabolized to stachyose and raffinose, which was confirmed by the detection of high levels of D-glucose and D-fructose in desiccation sensitive mutant seeds [21]. Similar results were obtained by Jing et al. [39], who found that overexpression of galactinol and raffinose synthases was associated with higher concentrations of galactinol, raffinose and stachyose in *Arabidopsis* mutants than in WT along with enhanced DT in developing mutant seeds. An increase in RFOs accumulation has been recently observed in the orthodox seeds of the Brazilian native tree *Erythrina speciosa* as well [40]. These results suggest that the increased expression of genes related to the raffinose pathway and accumulation of soluble nonreducing sugars in the cytoplasm during late seed maturation, among other things, might be one of the keys to obtaining DT in seeds. However, the exact molecular mechanisms underlying the transition of the cytoplasm into the glassy state remain unknown and probably involve a whole range of genes coding proteins of different functions.

Another strategy to reduce metabolic activity is to slowdown the machinery dedicated to metabolic control and respiratory processes. Leprince et al. [41] postulated that an important factor in obtaining DT is the reduction of ATP demand. In recently conducted transcriptomic and proteomic studies on seeds of three coffee species, Stavrinides et al. [12] showed that tested species shared a decrease in the expression of genes associated with energy production. The downregulated genes were related to mitorespiration (i.e., several units of ATP synthase, NADH dehydrogenase complex I, cytochrome bc1 complex, cytochrome c oxidase) and the tricarboxylic acid cycle (TCA; isocitrate dehydrogenase, pyruvate dehydrogenase, succinyl-CoA ligase, dihydrolipoyl dehydrogenase). Interestingly, comparative analysis showed some significant differences in the regulation of mitochondrial energy metabolism between desiccation-tolerant and desiccation-sensitive seeds. Seeds of the desiccation sensitive *Coffea canephora* displayed higher expression of the translocase inner membrane subunit (TIM44-2) and the mitochondrial splicing factor (OTP439), which are involved in basal cellular processes such as protein import and organelle posttranscriptional processes, respectively. On the other hand, desiccation tolerant seeds of *Coffea arabica* and *Coffea eugenioides* exhibited a down regulation of many genes crucial for the regulation of energetic processes, such as *mMDH1*, *VDAC1*, and *ATP5D*. This pattern suggests that DT seeds effectively slow the oxidative phosphorylation machinery [12] and seems to support the theory that reducing ATP demand is crucial for the acquisition of DT. The importance of reducing the expression level of the *VDAC* gene appears to be confirmed by the results of research conducted on elm (*Ulmus pumila* L.) seeds. Wang et al. [42] showed that overexpression of VDAC during controlled deterioration treatment was linked to an altered mitochondrial morphology. Moreover, the desiccation sensitive *C. canephora*, during seed late maturation, was characterized by increased transcription of several respiratory electron transfer chain complex compound genes involved in mitochondrial posttranscriptional and translational activities. Thus, recalcitrant seeds maintain high energy metabolism, which may be among the reasons for their DS. Another upregulated gene in desiccation-sensitive *C. canephora* was encoding formate dehydrogenase (FDH), a positive regulator of cell death, the defense response and hypoxia tolerance, metal toxicity, and low pH in *Arabidopsis* [12,43,44], and prohibitin (PHB3), which is associated with the nitric oxide-mediated stress response [12,45]. These transcriptional fingerprints translated into specific respiratory patterns. Upon desiccation, tolerant seeds displayed a stable reduction in their respiratory rate, while sensitive seeds experienced a drastic reduction, and at the same time, the respiration rate was higher than that measured for *C. arabica* and *C. eugenioides* [12]. Similar observations were made in *Castanea sativa* recalcitrant seeds. Desiccation sensitive cotyledons showed a higher respiration rate than more tolerant axes [41].

Uncoordinated slowdown of the metabolic machinery during desiccation leads to lipid oxidation, membrane damage and, consequently, to the death of DS seeds, which is associated with the accumulation of ROS (reactive oxygen species) [41,46]. Hence, genes associated with mitochondrial and respiration processes seem to play an important role in the development of DT. Wagner et al. [47] suggested that mitochondrial metabolism may be related to abscisic acid (ABA)-dependent transcriptional regulation. However, the exact genetic mechanisms of metabolic decreases in seeds remain unknown. Therefore, further research is needed to discover the genetic factors that make metabolism switch off in a coordinated and smooth way. To the best of our knowledge, the research carried out by Stavrinides et al. [12] is the only study that applies a wide transcriptomic and proteomic perspective with different types of seeds.

## 3. Antioxidative System and Regulation of the Redox State

The mechanisms of seed longevity and aging are still under intensive study. The main factor considered to have a huge impact on these processes is ROS [46,48,49,50,51]. During long-term seed storage, ROS accumulation leads to damage to lipids, DNA, and proteins and consequently contributes to decreased germination, loss of seed vigor, and even death [52,53,54]. It is believed that mitochondria are the main producers of ROS due to their important role as energy sources for cell growth and metabolism. Therefore, a pivotal role of mitochondria is metabolic shut down under seed storage conditions, as mentioned above. Nevertheless, there are active and passive mechanisms that are responsible for the detoxification of the ROS accumulated in the cytosol and cell compartments of developing and mature seeds. Active systems involve numerous proteins that allow seeds to wait out unfavorable conditions in a dormant state. Such specific antioxidant enzymes in seeds are superoxide dismutases, catalases, glutathione and ascorbate peroxidases, monodehydroascorbate, dehydroascorbate, and glutathione reductases [11,48,55]. Passive mechanisms include low molecular weight antioxidants, such as glutathione oxidation form (GSH) and glutathione disulfide form (GSSG)), tocopherol, and ascorbic acid (Asc).

Under stress, glutathione is maintained in the reduced state by action, and the accumulation of GSSG is often correlated with increased stress. Glutathione half-cell redox potential (E_GSSG/2GSH_) is an important indicator of the cell redox state and a specific marker of seed viability during desiccation stress [56]. Differences in half glutathione cells (E_GSSG/2GSH_) were observed in Norway maple (*Acer platanoides* L) orthodox seeds and in sycamore (*Acer pseudoplatanus* L.) recalcitrant seeds [52]. Thiol-disulfide transitions take part in cell signaling and control of catalytic activity, regulatory switches and protective mechanisms [57,58]. It is supposed that redox processes are involved in the metabolic regulation of maturing seeds and in the establishment of DT [59]. 

Regulation of the redox state can be controlled by antioxidant enzymes and compounds containing disulfide groups gluthation, peroxiredoxins (Prxs), thioredoxins (Trxs), and glutharedoxin (Grxs) [60,61]. This suggests that peroxiredoxin acts in redox-dependent cell signaling and can play an important role in defining the difference between seeds of the orthodox and recalcitrant types. Ratajczak et al. [52] showed that 1-Cys-Prx is reduced in Norway maple seeds (DT) and oxidized in sycamore seeds (DS) during development.

Moreover, other genes, such as *Prxs* and *Trxs*, have been reported as possible regulators of the redox state during different stages of seed development.

Peroxiredoxin family members have an important role in regulating and maintaining the redox balance in seed cells. It is also believed that these proteins prevent germination during stress conditions [62]. Prxs were first identified in yeast and are widely distributed among living organisms. Plant peroxiredoxins can be divided into four classes: typical 2-Cys Prx, atypical 2-Cys Prx, 1-Cys Prx, and Prx Q, located in distinct cell compartments including the chloroplast and mitochondrion [63]. Prxs are capable of reducing H_2_O_2_, alkyl hydroperoxides, and hydroxyl radicals [62]. They demonstrate antioxidant activity by protecting lipids, proteins, and DNA against ROS [64,65].

Prxs are intensively expressed during seed development, especially at stages associated with water loss during late maturation and in mature seeds in the dry state [66,67,68,69]. Therefore, it was primarily suggested that Prxs are involved in the maintenance of seed dormancy [64]. However, overexpression of barley 1-Cys Prx did not induce seed dormancy in transgenic *Arabidopsis* plants but inhibited germination under unfavorable conditions [62]. Furthermore, the expression level of the Prx in nondormant mutant *aba1* seeds was comparable with that of the WT [68], which also contradicts the hypothesis about the maintenance of dormancy. Interestingly, differences in Prx IIF transcript and protein levels as well as in the level of posttranslational modification have been observed between *A. platanoides* L. desiccation-tolerant seeds and *A*. *pseudoplatanus* L. desiccation-sensitive seeds during desiccation [52]. Thus, different expression patterns of peroxiredoxins may influence seed viability and longevity.

A similar conclusion was reached by Chen et al. [70] in an ectopic expression study of *Nelumbo nucifera* 1-Cys-Prx encoded by *NnPER1* gene in transgenic *Arabidopsis*. Over expression of *NnPER1* improved seed tolerance to adverse conditions by detoxifying ROS. However, the exact functions of Prxs in seeds remain to be discovered.

Another possible redox state regulator system involves the thioredoxin gene family. In higher plants, Trxs are divided into groups m, f, x, y, o and h based on their amino acid sequences. Connected with seeds are type h NADPH-dependent thioredoxins, which are most abundant in the mature state and in the nucleus of cells under oxidative stress [71]. Trxhs are crucial for the germination process by reducing storage proteins and mobilizing lipids [72,73,74]. However, an increased reduction in Trxhs during the late stages of seed development and germination under oxidative stress has been observed, suggesting, along with their localization in the nucleus, that Trxhs may be a part of the antioxidative system [71]. Furthermore, NADPH thioredoxin reductase (NTR) may be involved in the reduction of 1-Cys-Prx, thus suggesting that the NTR/Trx system indirectly influences ROS detoxification by peroxiredoxins [75]. Nevertheless, the role of Trxh in different types of seeds remains unclear.

## 4. Seed Coat as Defense Mechanism

The seed coat (testa) develops from the maternal tissue, the integuments, originally surrounding the ovule and acts as a physical and chemical barrier between the embryo and external conditions [11,23,29]. It is composed of several layers of specialized cell types. Its inner layer, endothelium, is rich in flavonoid compounds, which scavenge the reactive oxygen species and restraining oxidative stress [23,29]. Moreover, cell walls of palisade layer (PL) contain suberin, while the inner integument contain cutin, lipophilic polymers, create impermeable barrier to water gases and solutes [23,76,77]. Therefore, these polymer seed coat compounds might play a role in preventing oxidation stress and seed aging, by limiting the diffusion of atmospheric oxygen into the seed [23]. Research on 225 tree species conducted by Daws et al. [78] showed that desiccation sensitive seeds are indeed characterized by a thinner seed coat than desiccation tolerant. It has been hypothesized, that this feature is correlated with rapid germination, which is a result of selection pressure from vertebrate seed predators and fungi pathogens [79,80]. Therefore, the ability to germinate excluding the dormancy state may minimize the duration of exposure to predation and pathogens in tropical, humid regions where DS species are most common [78]. This hypothesis seems to be supported by the research conducted on *Arabidopsis* mutants seeds with thinner and more permeable testa comparing to the WT, which germinated faster than the wild type [81].

Seed coat thickness and mechanical strength is associated with flavonoid compounds, mainly proanthocyanidins (PAs), polysaccharides, and polyesters such as lignin and suberin. Some attempts to identify key genes regulating seed longevity connected to seed coat defense mechanisms have already been made. Righetti et al. [22] in the coexpression network of *M. truncatula* and *Arabidopsis* analysis found that *wrky3* and *nfxl1* mutants seeds were affected with reduced longevity and higher testa permeability. WRKY3 transcription factor (TF) is highly expressed during last stages of seed development. Its deficiencies in mutant plants lead to more severe disease symptoms of fungal *Botrytis cinerea* infection [82]. Furthermore overexpression of grape (*Vitis labrusca*) WRKY3 in transgenic *Arabidopsis* resulted in improved resistance to *Golovinomyces cichoracearum*, plants also showed improved salt and drought stress tolerance during the germination, seedling and the mature plant stages [83]. Second transcription factor encoded by *NFXL1* has been linked to trichothecene phytotoxin-induced response in *Arabidopsis* and higher resistance to abiotic stresses such as salt, drought and high light intensity [84,85], but also represses *Fusarium graminearum* fungus resistance in wheat [86]. Target genes for these TFs are yet to be determined.

Probably peroxidases are involved in biosynthetic pathway of the flavonoids, however no specific genes associated with polymerization of these compounds have been identified yet [23]. During seed desiccation polymeric flavonoids are being oxidized by the laccase-type polyphenol oxidase Testa Glabra 10 (TT10) and as a result become brown pigments [87,88]. Mutations in *TT10*, manifested by disturbed testa pigmentation, lead to reduced dormancy and longevity [89]. For example, rapeseed (*Brassica napus*) and flax (*Linum usitatissimum*) yellow seed mutants showed greater reductions in germination in comparison with dark-pigmented seeds subjected to accelerated aging [90,91].

Another factor that affects testa strength and waterproof is lignin, which accumulates in cell walls of seed coat integuments [92,93]. This polymer of monolignol has been associated with permeability and resistance to mechanical damage in soybean [94] and is thought to act as antioxidant [90]. It has been shown, that Testa Transparent 10 participates in seed coat lignin biosynthesis in *Arabidopsis* and rapeseed [11]. *tt10* mutants are characterized by reduced lignin content in the seed coat and their longevity during natural aging is being affected [81,95]. 

Suberin is another biopolymer involved in seed coat permeability. Several enzymes involved in suberin biosynthesis have been already described, including glycerol-3-phosphate acyl-transferases (GPAT), which encode transferases involved in the synthesis of acylglycerol precursors of suberin polymer [96,97,98]. Analysis of *gpat5 Arabidopsis* mutant revealed a reduction of suberin content in the seed coat [99]. Recently, Renard et al. [23] linked peroxidases with biosynthesis probably of both lignin and suberin in the testa. *Arabidopsis prx2* and *prx25* loss of function mutants where characterized by higher permeability than the WT seeds [23]. Moreover, overexpression of COG1 and ATHB25 transcription factors causes the increase in suberin accumulation in the palisade layer, which results in higher resistance to seed aging [24,100]. However, target genes for these TFs have not been identified yet, but it has been observed that *cog1-2D* and *athb25-1D* mutants accumulate more ABA [23]. Interestingly, MYB41 TF in *Arabidopsis thaliana* and *Nicotiana benthamiana* activates aliphatic suberin synthesis and deposition steps in the ABA-dependent manner [101], thus COG1 and ATHB25 my indirectly influence this TF. As Renard et al. [23] hypothesize, COG1 and ATHB25 may directly regulate suberin biosynthetic genes, such as glycerol-3-posphate acyltransferases, cinnamyl alcohol dehydrogenases, fatty acyl CoA reductases, and laccases. On the other hand, peroxidases PRX2 and PRX25 may be indirectly regulated by COG1 TF through the upregulation of Gibberellin 3-Oxidase 3 (GA3OX3) [23,24]. GA3OX3 converts the inactive gibberellin (GA) precursors, GA9 and GA20, in the bioactive gibberellins GA4 and GA1 [102]. Additionally, ATHB25 in *Arabidopsis* positively regulates another gene involved in GA biosynthesis, Gibberellic Acid Oxidase 2 (GA3OX2), probably in the indirect way through upregulation of another TF, which have not been identified yet [100]. GAs have been shown to contribute to the formation of seed coat through the induction of starch degradation at the epidermis and palisade, and by increasing mucilage synthesis at the epidermis [24,103]. It is also possible that GA may influence seed longevity by the induction protoanthocyanidin synthesis in the endothelial layer of the seed coat [29,100].

Seed tolerance to adverse conditions has been also linked to the light perception. Park et al. [104] suggested that COG1 functions as a negative regulatory component within phytochromes PHYA (far-red light receptor) and PHYB (red light receptor) signaling pathways. Overexpression of COG1 causes defects in PHYA- and PHYB mediated light responses [104]. More recent research of *phyA* and *phyB* loss of function *Arabidopsis* mutants showed, that red and far-red light negatively influence the seed tolerance to unfavorable conditions [24]. Both mutants produced more tolerant seeds with reduced permeability to tetrazolium and increased suberin content. An inverse relationship was observed regarding cryptochrome (*cry1*, *cry2*) blue light receptors mutants. Produced seeds were sensitive to aging, seed coat was more permeable, and the suberin content was reduced [24]. This mechanism may be correlated with GA action, since light inhibits GA biosynthesis [24,105].

## 5. Late Embryogenesis Abundant (LEA) Proteins Accumulation

Late embryogenesis abundant (LEA) proteins form a group of several dozen, mostly hydrophilic, rich in Gly and small amino acids such as Ala and Ser, molecules ranging in weight from 10 to 30 kDa and above [106,107]. Depending on the plant species, they can be categorized into several groups characterized by specific amino acid motifs: LEA1-LEAn, dehydrins and seed maturation proteins (SMPs) [108]. LEAs were first described in cottonseeds during their late maturation stages of development and germination [109]. Further research showed that these proteins are widely distributed in the plant kingdom and have been identified from algae, mosses, ferns, to angiosperms and resurrection plants [108,110]. Interestingly, LEA-like proteins are also found in a variety of organisms, such as anhydrobiotic nematodes [111,112], brine shrimp [113], rotifers [114,115], and some bacterial species [108]. Although LEAs were first described in embryonic tissues, thirty years of intensive research showed that the expression of LEA genes is also significantly induced in vegetative organs (i.e., callus, flowers, roots, leaves, buds) under abiotic stresses such as desiccation, salinity, and cold [116,117,118,119,120].

The in vivo activities of most LEA proteins remain unknown. However, based on some research on transgenic plants, it is considered that they have a protective function and play an important role in developing desiccation tolerance in seeds. For example, silencing of the three LEA proteins from group 4 in *Arabidopsis thaliana* was enough to cause water deficit sensitivity [121]. Moreover, studies on recalcitrant seeds of *Avicennia marina* in comparison to orthodox seeds showed the absence of LEA proteins in desiccation-sensitive seeds, which led authors to the conclusion that this might be one of the reasons for DS [122]. In addition, the group 3 LEA protein HVA1 from *Hordeum vulgare* increased plant tolerance to water deficit when incorporated into rice plants. Transgenic plants also maintained higher growth rates than the control group and showed better recovery when the stress conditions were removed [123]. Additionally, there are some evidences, based on invertebrate studies, that at least some LEA proteins require the presence of trehalose for full protective action during desiccation, heat and freeze stresses [124,125,126,127]. Li et al. [128] observed connection between overexpression of the trehalose-6-phosphate synthase gene (*TPS1*) from rice, higher accumulation of LEA14A and dyhydrin DHN6, and improved tolerance of rice seedling to cold, high salinity and drought treatments. Nevertheless, the importance of trehalose occurrence regarding LEA proteins in seeds is still elusive.

More recent studies seem to confirm that the accumulation of specific LEA proteins during seed development might be the key to obtaining DT. Analysis of the transcriptome and proteome of intermediate coffee seeds revealed that 15 seed-specific LEA genes were massively transcribed and translated during the late maturation stage. In comparison with orthodox seeds, the authors did not observe any specific lack of these proteins [129]. Other studies on late maturation in coffee seeds showed significantly higher expression levels and protein contents of one specific LEA, EM6, in *C. arabica* and *C. eugenioides*, which show intermediate desiccation tolerance in comparison with the recalcitrant *C. canephora* [12]. Similar observations were made by Delahaie et al. [25] in the juxtaposition of desiccation-sensitive *Castanospermum australe* and desiccation-tolerant *Medicago truncatula*. Comparative proteomic analysis showed that six LEA polypeptides, SBP65, MP2, PM25, LEAm, EM1, and the abovementioned EM6, were less abundant in desiccation-sensitive *C. australe* [25]. EM6 plays a crucial role in water binding during *Arabidopsis* seed maturation. The absence of this protein may affect the stability of the glassy state of the cytoplasm, caused by increased water absorption, and thus may lead to the disintegration of membrane structures [130]. This acting as a molecular sponge protein may participate in water loss control during seed maturation and drying [12].

In transcriptomic studies on recalcitrant tea seeds, all LEA proteins were found to be downregulated. Therefore, Jin et al. [131] speculated that an insufficient level of transcription of these genes results in sensitivity to dehydration. On the other hand, research on desiccation-sensitive *C. australe* seeds showed high accumulation of two dehydrins: BudCar5 and DHN-cognate, which were barely detected in desiccation tolerant *M. truncatula*. Furthermore, in *C. australe*, dehydrins constitute 83% of the LEA proteome, while in *M. truncatula*, dehydrins constitute only 20% [25]. Moreover, silencing dehydrins in *Arabidopsis* does not affect DT, nor does the lack of one or two LEA genes [129]. These results suggest that not all LEA proteins are involved in the acquisition of DT. There must be a more complex relationship that contributes to DT in seeds, which involves specific LEA proteins and regulatory factors. Most of these genes are under ABA-dependent signaling control; moreover, the promoters of LEA genes show *cis*-element responses [132,133]. Therefore, further research on the expression mechanisms of LEA genes in different types of seeds is needed.

## 6. Gene Expression Regulation at the Transcriptomic Level

The mechanisms that regulate gene expression have a huge impact on the plant response to stress. The direction of transcriptional reprogramming largely affects whether the reaction to adverse conditions will end in desiccation tolerance or sensitivity. The response to water limitation and other stresses is mainly regulated by ABA signaling, but there are also mechanisms independent of this factor [134,135]. In the case of vegetative tissues, many transcription factors (TFs) have already been shown to induce gene expression associated with defense mechanisms. For example, the WRKY TF induces synthesis of raffinose family oligosaccharides in *Boea hygrometrica*, which leads to the transition of the cytoplasm into the glassy state [136]. Overexpression of MYB10 from the resurrection plant *Craterostigma plantagineum* in transgenic *Arabidopsis* resulted in increased tolerance to drought [137]. Some studies have already shown that there are some differences in gene expression patterns between desiccation-tolerant and desiccation-sensitive seeds during their maturation [12,21]. However, little is known about the mechanisms that regulate the acquisition of DT at the gene expression level. It is widely accepted that seed development is mainly under ABA signaling control, which involves many TFs during maturation and seed dormancy [138,139].

ABA plays a pivotal role in stress resistance in plants through the regulation of physiological processes that participate in seed lipid and storage protein synthesis and is crucial for the acquisition of DT and dormancy in seeds. ABA hormone signaling is associated with the expression of regulatory genes involved in seed maturation [140,141]. This pathway is composed of PYR/PYL-RCAR receptors, PP2C phosphatases, and SnRK2 kinases and works through the regulation of the expression of genes that contain the ABSCIC ACID RESPONSE ELEMENT (ABRE) motif in their promoter region (reviewed by [134,142]).

It is commonly believed that the AFL subfamily of B3 TFs plays a crucial role in ABA signaling during seed development and maturation. The name of this group of genes—AFL—comes from the first letters of the genes, which are part of this subfamily: ABA INSENSITIVE 3 (*ABI3*), FUSCA3 (*FUS3*), and LEAFY COTYLEDON 2 (*LEC2*). Additionally, AFL also includes LEAFY COTYLEDON 1 (*LEC1*), which is an ortholog of the NF-YB subunit of the CCAAT-binding TF [143,144,145,146]. These master TFs control the expression of thousands of other genes involved in all seed development stages. The main feature of AFL TFs is the presence of a B3 DNA-binding domain, which consists of a seven-stranded β-sheet arranged in an open barrel that is accompanied by two short α-helicases located at the two ends of the barrel [147]. The B3 domain binds to the RY motifs and activates maturation-specific genes; additionally, ABI3 activity requires another domain, which binds to ABA response elements [148,149,150,151,152]. Mutations in AFL genes cause shortages of reserve material in seeds, lower the degree of DT and cause difficulties in the acquisition of dormancy [153,154,155,156]. In this review, we will focus only on the major regulators of transcription in developing and maturing seeds, representing B3 transcription factors; for a wide review of LEAFY COTYLEDON 1, see Jo et al. [157].

**Figure 1:**
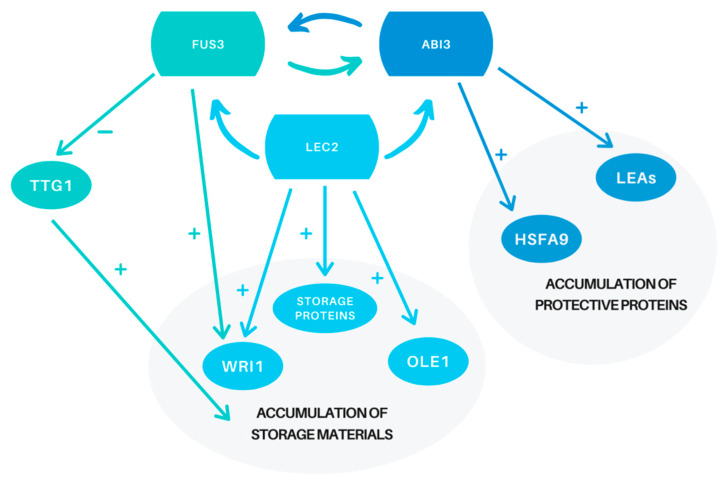
Simplified diagram illustrating the effect of B3 transcription factors during the maturation of seeds. a.—FUS3 represses TRANSPARENT TESTA GLABRA 1 (TTG1) transcription factor (TF), which is a negative regulator of genes related to fatty acid and storage protein biosynthesis, and positively regulates WRINKLED 1 (WRI1), an inducer of fatty acid biosynthesis; thus, FUS3 indirectly positively affects the accumulation of storage materials. FUS3 also regulates ABI3 expression in the lateral parts of cotyledons. b.—LEC2 regulates other B3 transcription factors—FUS3 and ABI3, preventing anthocyanin and chlorophyll accumulation and by positive regulation of WRI1 and OLE1 takes part in intensified fatty acid biosynthesis and storage; LEC2 also positively regulates the expression of 2S and 12S storage proteins. c.—ABI3 regulates expression of FUS3 in the embryo axis and cotyledons and indirectly takes part in the accumulation of heat shock protective proteins by positive regulation of HSFA9 TF; ABI3 is a master regulator of late embryogenesis abundant (LEA) protective proteins.

The first plant B3 TF identified was VIVIPAROUS-1 (VP1) in maize, which is an ortholog of the *Arabidopsis* ABI3 transcription factor. Maize *vp1* mutant seeds are ABA insensitive and as a result are unable to reach a quiescent state, which is manifested by the germination of seeds that are still in the corn cob [153]. Analogously, Arabidopsis *abi3* mutant seeds are unable to complete the maturation program; as a result, they do not acquire dormancy and are characterized by a low degree of DT [158]. As Sano et al. reviewed [11], some *abi3* mutant seeds remain green due to defective chlorophyll catabolism, resulting in reduced longevity and storability. Hence, ABA INSENSITIVE 3 must be involved in the reduction of anthocyanin and chlorophyll accumulation. Coexpression network studies on *Medicago* and *Arabidopsis* revealed that ABI3 shows the highest number of correlations with DT-related genes [22]. Thus, this TF seems to be a crucial transcriptional regulator during seed development. Some mechanisms that involve ABI3 during the maturation program have already been described. For example, induction of seed dormancy and desiccation tolerance in *Arabidopsis* is indirectly correlated with ABI3 by regulation of seed-specific heat shock factor HSFA9. Positive regulation of this TF results in the accumulation of protective heat shock proteins [159,160,161,162,163,164], which improve seed thermo tolerance and resistance to controlled deterioration treatment (CDT) [165]. Verdier et al. [166] came to similar conclusions with a coexpression gene regulatory network analysis in *M*. *truncatula* seeds. ABI3 also controls the expression of seed-specific aquaporin genes: tonoplast intrinsic proteins TIP3-1 and TIP3-2, which have been associated with seed longevity [167]. Furthermore, Delahaie et al. [25] linked the lack of DT in *Medicago abi3* mutants to reduced accumulation of LEA proteins. Similarly, more than half of LEA genes involved in acquiring DT in seeds are misregulated in *Arabidpsis abi3* mutants [168]. ABA INSENSITIVE 3 in *A*. *thaliana* is not only a positive regulator of LEAs involved in DT acquisition (Figure 1) but is also a repressor of LEAs specific for vegetative tissues [168]. To et al. [158], in the expression analysis of *lec2*, *abi3*, and *fus3 Arabidopsis* mutants, suggested that ABI3 regulates another AFL TF, FUS3, specifically in the embryo axis and cotyledons. Moreover, ABI3 is regulated by other AFLs, including itself [158].

Braybrook et al. [148] proved that LEC2 is expressed at the earliest stages of seed development and remains active until the middle phase of maturation. This protein directly and indirectly activates the expression of genes involved in seed maturation and the accumulation of lipids and seed storage proteins (SSPs) by inducing the expression of other AFL TFs, such as ABI3 and FUS3 [148,169]. LEC2 is required in the early stages of seed development to activate FUS3 expression and in later stages of maturation to maintain the expression of ABI3 at a stable level [158]. By positive regulation of FUS3 and ABI3, LEC2 also prevents anthocyanin and chlorophyll accumulation [149,158]. Straightway, LEC2 regulates the expression of WRINKLED 1 (WRI1), which plays a crucial role in fatty acid biosynthesis during seed maturation (Figure 1). Interestingly, WRI1 seems to be transcriptionally induced by LEC2 only in the hypocotyl of the embryo [170]. Another gene involved in the accumulation of storage reserves regulated by LEC2 is *OLE1*, encoding oleosin [148,171], and genes encoding 2S and 12S storage proteins [148,149,154] (Figure 1). Therefore, mutations in *LEC2* may cause a reduction in the amount of reserve materials in seeds. Indirectly, however, LEC2 affects the expression of LEA *EM1* and *EM6* genes by inducing the expression of the EEL basic leucine zipper (bZIP) TF [148], which is a negative regulator of those EM proteins in *Arabidopsis* [172]. The EEL TF competes with ABI5, which is a positive regulator of EMs, for their promoter sites [172]. In the later stages of the maturation program, during drying when LEC2 is present in smaller quantities, the EEL volume also drops, and the expression of the DT-related proteins EM1 and EM6 is induced by ABI5. Therefore, the main role of LEC2 in the acquisition of DT in seeds seems to be the regulation of genes involved in the accumulation of storage materials.

In the case of the accumulation of storage materials in developing seeds, another crucial B3 transcription factor, FUSCA3, is involved. Wang and Perry [173], in the expression analysis in *Arabidopsis,* identified that direct and indirect target genes for FUS3 are associated with nutrient reservoir activity, lipid localization, storage, metabolic processes, and seed oil body biogenesis. Indirect transcriptional control of genes related to storage materials by FUS3 is linked with negative regulation of TRANSPARENT TESTA GLABRA 1 (TTG1), which is a TF that suppresses the accumulation of seed oil and storage proteins in *Arabidopsis* [174] (Figure 1). Suppression of TTG1 by FUS3 may lead to the promotion of four out of five genes encoding precursors of 2S storage proteins [174]. Moreover, *ttg1* mutants are characterized by a dramatic accumulation of seed storage reserves, including storage proteins and fatty acids [175]. TTG1 is also associated with the accumulation of anthocyanins, which compete for the same carbon source as fatty acid biosynthesis processes in maturing seeds. Moreover, anthocyanins repress the expression of genes encoding pivotal reductases involved in the elongation of the fatty acid carbon chain [173,174]. Additionally, together with LEC2, FUS3 induces the expression of WRI1, positive regulator of fatty acid biosynthesis compounds in developing seeds [176] (Figure 1). Thus, by repressing TTG1 expression and enhancing WRI1, FUS3 indirectly promotes the biogenesis of storage lipids. In addition to a significant role in the accumulation of storage materials, FUS3 together with AKIN10 kinase in *Arabidopsis* probably plays an important role in the establishment and maintenance of seed dormancy, as well as in the regulation of lateral organ development [177]. Finally, analysis of the *Arabidopsis fus3* mutant showed that FUSCA3 is involved in the regulation of ABI3 expression in the lateral parts of cotyledons [158].

LEC transcription factors activity is strictly correlated with plant hormones, the germination inhibitor abscisic acid and growth promoter gibberellic acid, which are important factors in controlling seed maturation, germination, and seedling growth [178,179,180]. 

ABA plays a critical role in the induction and the maintenance of seed dormancy and inhibits the transition from embryonic to germinative growth [181,182]. Its accumulation increases during initiation of maturation phase, remains high until the late phase of maturation, when it starts to decline to finally reach very low levels in developing seedlings [179,180,183]. ABA promotes seed maturation and dormancy, by stimulation of FUS3 and LEC1 TFs, which positively regulate the biosynthesis of storage materials [180,184]. Furthermore, FUS3 expression is being enhanced by exogenously introduced ABA [184] and FUS3 induces the increase of ABA as well [185]. Thus, FUS3 and ABA act as positive regulators to each other [180].

Gibberellins, on the other hand, are necessary for promotion of seed germination [182,186]. Therefore GA levels are low during seed maturation phase and increase through germination [180,183,186]. The level of GA is regulated by FUS3 and LEC2 TFs, which repress the enzymes involved in transformation of GA into the active form [187]. Both LEC2 and FUS3 downregulate GA biosynthesis gene *GA3OX2* [187], additionally FUS3 negatively regulates *GA3OX1* [185]. Whereas when it comes to the seedlings, LEC TFs have been shown to be down regulated by GA [188,189,190].

Master regulators of seed development and maturation have been extensively studied in model organisms, such as *Arabidopsis*, legumes, and maize. However, there is still not enough data on differences in the expression patterns of these factors in different types of seeds of other species. An attempt was made by Stavrinides et al. [12], who studied whether there are differences in the expression patterns of TFs between intermediate and recalcitrant coffee seeds during late maturation. Interestingly, very few TFs were identified in the transcriptome of intermediate coffee seeds [12]. Most DT-related regulatory genes were implicated in RNA processing or chromatin remodeling. In contrast, an inverse relationship was identified in the desiccation-sensitive seeds, which did not contain chromatin remodeling factors, but many TFs related to developmental activities and cell differentiation. The authors did not detect differences in expression patterns of master ABA-dependent TFs but reported changes in regulation of other genes encoding proteins that play important roles in ABA signaling. DT-related were positive regulators of ABA-mediated seed germination inhibition in *Arabidopsis*–NHL6 [12,191], epistatic to ABI5–AFP2 [12,192] and ABO5, which probably positively regulates the expression of stress-inducible genes [12,193]. Specifically, PYL8 was upregulated in recalcitrant seeds. Overexpression of this gene in *Arabidopsis* leads to ABA hypersensitivity in seeds and boosts the level of seed dormancy [194]. Stavrinides et al. [12] concluded that ABA sensitivity may be the key to regulating DT acquisition in seeds, as mitochondria were recently shown to slow down metabolic activity in response to ABA. Nevertheless, little is known about the differences in the transcription regulatory processes in desiccation-tolerant and -sensitive seeds during development and maturation.

## 7. Conclusions

**Figure 2:**
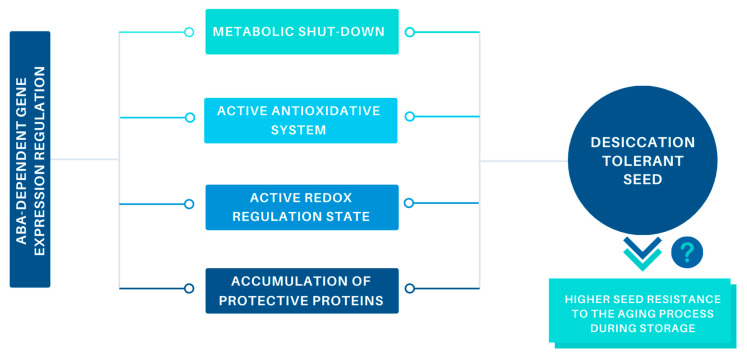
Simplified schema showing what factors are necessary for the development of desiccation tolerance in seeds. During seed maturation, abscisic acid (ABA)-dependent signaling affects gene expression patterns, which lead to coordinated slowing of metabolism, activation of antioxidative and redox regulation systems, and accumulation of protective proteins. The acquisition of desiccation tolerance may contribute to higher seed resistance to the aging processes during long-term storage.

In this review, we have summarized findings about the genetic basis for acquiring desiccation tolerance (DT) and longevity in seeds. This process is very complicated and involves numerous genes encoding proteins involved in gene expression, metabolic shutdown and storage material accumulation. Furthermore, there are differences in expression patterns during seed maturation between desiccation-tolerant and recalcitrant types of seeds. It appears that a smooth metabolic shutdown, accumulation of protective LEA proteins, and efficient antioxidative systems are crucial for the acquisition of DT, dormancy and longevity (Figure 2). All of these processes seem to be under abscisic acid (ABA)-dependent regulation, which includes the master transcription factors (TFs) ABI3, FUS2, and LEC2. Although TFs appear to coordinate the maturation program in every type of seed, there are some differences in expression patterns between desiccation-tolerant and -sensitive seeds. The reasons for these differences, however, remain unclear. We believe that influencing ABA sensitivity may enable us to control the acquisition of DT in recalcitrant seeds. Such knowledge would help improve crop yields and storage conditions in gene banks. Although many genes associated with DT have been identified, little is known about the mechanisms that control seed DT, dormancy and longevity. Comparative studies between orthodox, recalcitrant, and intermediate seeds are needed to explain why differences in DT levels occur. 

Searching for answers concerning the operation of the entire mechanism affecting tolerance to seed desiccation will allow us to answer the following important questions: why seeds are aging and whether genes responsible for DT also reduce the aging process of these seeds.

## References

[1] Ghassemi-Golezani K., Chadordooz-Jeddi A., Zehtab-Salmasi S. (2015). Effects of seed size and aging on field performance of lentil (*Lens culinaris* Medik.) under different irrigation treatments. Acta Agric. Slov..

[2] Ghassemi-Golezani K., Ghanehpoor S., Mohammadi-Nasab A.D. (2009). Effects of water limitation on growth and grain filling of faba bean cultivars. J. Food Agric. Environ..

[3] Han F., Chen H., Li X.-J., Yang M.-F., Liu G.-S., Shen S.-H. (2009). A comparative proteomic analysis of rice seedlings under various high-temperature stresses. BBA Proteins Proteom..

[4] Leprince O., Pellizzaro A., Berriri S., Buitink J. (2016). Late seed maturation: Drying without dying. EXBOTJ.

[5] Hay F.R., Probert R.J. (2013). Advances in seed conservation of wild plant species: A review of recent research. Conserv. Physiol..

[6] Groot S.P.C., Surki A.A., de Vos R.C.H., Kodde J. (2012). Seed storage at elevated partial pressure of oxygen, a fast method for analysing seed ageing under dry conditions. Ann. Bot..

[7] Bajaj Y.P.S. (2013). Cryopreservation of Plant Germplasm I.

[8] Tweddle J.C., Dickie J.B., Baskin C.C., Baskin J.M. (2003). Ecological aspects of seed desiccation sensitivity. J. Ecol..

[9] Sershen N., Perumal A., Varghese B., Govender P., Ramdhani S., Berjak P. (2014). Effects of elevated temperatures on germination and subsequent seedling vigour in recalcitrant *Trichiliaemetica seeds*. S. Afr. J. Bot..

[10] Redden R.J., Yadav S.S., Maxted N., Dulloo M.E., Guarino L., Smith P. (2015). Crop Wild Relatives and Climate Change.

[11] Sano N., Rajjou L., North H.M., Debeaujon I., Marion-Poll A., Seo M. (2016). Staying alive: Molecular aspects of seed longevity. Plant Cell Physiol..

[12] Stavrinides A.K., Dussert S., Combes M.-C., Fock-Bastide I., Severac D., Minier J., Bastos-Siqueira A., Demolombe V., Hem S., Lashermes P. (2019). Seed comparative genomics in three coffee species identify desiccation tolerance mechanisms in intermediate seeds. J. Exp. Bot..

[13] Li D.-Z., Pritchard H.W. (2009). The science and economics of ex situ plant conservation. Trends Plant Sci..

[14] Pardey P.G., Koo B., Wright B.D., Van Dusen M.E., Skovmand B., Taba S. Costing the Ex Situ Conservation of Genetic Resources: Maize and Wheat at CIMMYT. https://ageconsearch.umn.edu/record/97509.

[15] Walters C., Pammenter N.W., Berjak P., Crane J. (2001). Desiccation damage, accelerated ageing and respiration in desiccation tolerant and sensitive seeds. Seed Sci. Res..

[16] Marques A., Nijveen H., Somi C., Ligterink W., Hilhorst H. (2019). Induction of desiccation tolerance in desiccation sensitive *Citrus limon* seeds. J. Integr. Plant Biol..

[17] Alpert P. (2005). The Limits and frontiers of desiccation-tolerant life. Integr. Comp. Biol..

[18] Hoekstra F.A., Golovina E.A., Buitink J. (2001). Mechanisms of plant desiccation tolerance. Trends Plant Sci..

[19] Oliver M.J., Tuba Z., Mishler B.D. (2000). The evolution of vegetative desiccation tolerance in land plants. Plant Ecol..

[20] Mishler B.D., Churchill S.P. (1985). Transition to a land flora: Phylogenetic relationships of the green algae and bryophytes. Cladistics.

[21] González-Morales S.I., Chávez-Montes R.A., Hayano-Kanashiro C., Alejo-Jacuinde G., Rico-Cambron T.Y., de Folter S., Herrera-Estrella L. (2016). Regulatory network analysis reveals novel regulators of seed desiccation tolerance in *Arabidopsis thaliana*. Proc. Natl. Acad. Sci. USA.

[22] Righetti K., Vu J.L., Pelletier S., Vu B.L., Glaab E., Lalanne D., Pasha A., Patel R.V., Provart N.J., Verdier J. (2015). Inference of longevity-related genes from a robust coexpression network of seed maturation identifies regulators linking seed storability to biotic defense-related pathways. Plant Cell.

[23] Renard J., Martínez-Almonacid I., Sonntag A., Molina I., Moya-Cuevas J., Bissoli G., Muñoz-Bertomeu J., Faus I., Niñoles R., Shigeto J. (2020). PRX2 and PRX25, peroxidases regulated by COG1, are involved in seed longevity in *Arabidopsis*. Plant Cell Environ..

[24] Bueso E., Muñoz-Bertomeu J., Campos F., Martínez C., Tello C., Martínez-Almonacid I., Ballester P., Simón-Moya M., Brunaud V., Yenush L. (2016). *Arabidopsis* COGWHEEL1 links light perception and gibberellins with seed tolerance to deterioration. Plant J..

[25] Delahaie J., Hundertmark M., Bove J., Leprince O., Rogniaux H., Buitink J. (2013). LEA polypeptide profiling of recalcitrant and orthodox legume seeds reveals ABI3-regulated LEA protein abundance linked to desiccation tolerance. J. Exp. Bot..

[26] Berjak P., Pammenter N. (2013). Implications of the lack of desiccation tolerance in recalcitrant seeds. Front. Plant Sci..

[27] Obroucheva N., Sinkevich I., Lityagina S. (2016). Physiological aspects of seed recalcitrance: A case study on the tree Aesculus hippocastanum. Tree Physiol..

[28] Berjak P., Dini M., Pammenter N.W. (1984). Possible mechanisms underlying the differing dehydration responses in recalcitrant and orthodox seeds: Desiccation-associated subcellular changes in propagules of Avicennia marina. Seed Sci. Technol..

[29] Rajjou L., Debeaujon I. (2008). Seed longevity: Survival and maintenance of high germination ability of dry seeds. Comptes Rendus Biol..

[30] Walters C., Wheeler L.M., Grotenhuis J.M. (2005). Longevity of seeds stored in a genebank: Species characteristics. Seed Sci. Res..

[31] Pukacka S., Ratajczak E., Kalemba E. (2009). Non-reducing sugar levels in beech (*Fagus sylvatica*) seeds as related to withstanding desiccation and storage. J. Plant Physiol..

[32] Buitink J. (2000). Biological Glasses: Nature’s Way to Preserve Life.

[33] Gechev T.S., Dinakar C., Benina M., Toneva V., Bartels D. (2012). Molecular mechanisms of desiccation tolerance in resurrection plants. Cell. Mol. Life Sci..

[34] Peters S., Mundree S.G., Thomson J.A., Farrant J.M., Keller F. (2007). Protection mechanisms in the resurrection plant *Xerophyta viscosa* (Baker): Both sucrose and raffinose family oligosaccharides (RFOs) accumulate in leaves in response to water deficit. J. Exp. Bot..

[35] Cooper K., Farrant J.M. (2002). Recovery of the resurrection plant *Craterostigma wilmsii* from desiccation: Protection versus repair. J. Exp. Bot..

[36] Scott P. (2000). Resurrection Plants and the Secrets of Eternal Leaf. Ann. Bot..

[37] Ingram J., Chandler J.W., Gallagher L., Salamini F., Bartels D. (1997). Analysis of cDNA clones encoding sucrose-phosphate synthase in relation to sugar interconversions associated with dehydration in the resurrection plant *Craterostigma plantagineum* Hochst. Plant Physiol..

[38] Ghasempour H.R., Gaff D.F., Williams R.P.W., Gianello R.D. (1998). Contents of sugars in leaves of drying desiccation tolerant flowering plants, particularly grasses. Plant Growth Regul..

[39] Jing Y., Lang S., Wang D., Xue H., Wang X.-F. (2018). Functional characterization of galactinol synthase and raffinose synthase in desiccation tolerance acquisition in developing *Arabidopsis seeds*. J. Plant Physiol..

[40] Hell A.F., Kretzschmar F.S., Simões K., Heyer A.G., Barbedo C.J., Braga M.R., Centeno D.C. (2019). Metabolic changes on the acquisition of desiccation tolerance in seeds of the brazilian native tree *Erythrina speciosa*. Front. Plant Sci..

[41] Leprince O., Buitink J., Hoekstra F.A. (1999). Axes and cotyledons of recalcitrant seeds of *Castanea sativa* Mill. exhibit contrasting responses of respiration to drying in relation to desiccation sensitivity. J. Exp. Bot..

[42] Wang Y., Li Y., Xue H., Pritchard H.W., Wang X. (2015). Reactive oxygen species-provoked mitochondria-dependent cell death during ageing of elm (*Ulmus pumila* L.) seeds. Plant J..

[43] Choi D.S., Kim N.H., Hwang B.K. (2014). Pepper Mitochondrial FORMATE DEHYDROGENASE1 regulates cell death and defense responses against bacterial pathogens. Plant Physiol..

[44] Lou H.Q., Gong Y.L., Fan W., Xu J.M., Liu Y., Cao M.J., Wang M.-H., Yang J.L., Zheng S.J. (2016). A Formate dehydrogenase confers tolerance to aluminum and low pH. Plant Physiol..

[45] Wang Y., Ries A., Wu K., Yang A., Crawford N.M. (2010). The *Arabidopsis* Prohibitin gene PHB3 functions in nitric oxide-mediated responses and in hydrogen peroxide-induced nitric oxide accumulation. Plant Cell.

[46] Ratajczak E., Małecka A., Bagniewska-Zadworna A., Kalemba E.M. (2015). The production, localization and spreading of reactive oxygen species contributes to the low vitality of long-term stored common beech (*Fagus sylvatica* L.) seeds. J. Plant Physiol..

[47] Wagner S., Van Aken O., Elsässer M., Schwarzländer M. (2018). Mitochondrial energy signaling and its role in the low-oxygen stress response of plants. Plant Physiol..

[48] Bailly C. (2004). Active oxygen species and antioxidants in seed biology. Seed Sci. Res..

[49] Pukacka S., Ratajczak E. (2007). Age-related biochemical changes during storage of beech (*Fagus sylvatica* L.) seeds. Seed Sci. Res..

[50] Rajjou L., Lovigny Y., Groot S.P.C., Belghazi M., Job C., Job D. (2008). Proteome-wide characterization of seed aging in *Arabidopsis*: A comparison between artificial and natural aging protocols. Plant Physiol..

[51] Parkhey S., Naithani S.C., Keshavkant S. (2012). ROS production and lipid catabolism in desiccating *Shorea robusta* seeds during aging. Plant Physiol. Biochem..

[52] Ratajczak E., Małecka A., Ciereszko I., Staszak A.M. (2019). Mitochondria are important determinants of the aging of seeds. IJMS.

[53] Osborne D.J. (2000). Hazards of a germinating seed: Available water and the maintenance of genomic integrity. Isr. J. Plant Sci..

[54] Kranner I., Minibayeva F.V., Beckett R.P., Seal C.E. (2010). What is stress? Concepts, definitions and applications in seed science. New Phytol..

[55] Jeevan Kumar S.P., Rajendra Prasad S., Banerjee R., Thammineni C. (2015). Seed birth to death: Dual functions of reactive oxygen species in seed physiology. Ann. Bot..

[56] Kranner I., Birtić S., Anderson K.M., Pritchard H.W. (2006). Glutathione half-cell reduction potential: A universal stress marker and modulator of programmed cell death? Free Radic. Biol. Med..

[57] Dietz K.J., Hell R. (2015). Thiol switches in redox regulation of chloroplasts: Balancing redox state, metabolism and oxidative stress. Biol. Chem..

[58] Go Y.-M., Jones D.P. (2013). Thiol/disulfide redox states in signaling and sensing. Crit. Rev. Biochem. Mol. Biol..

[59] Colville L., Kranner I. (2016). Desiccation tolerant plants as model systems to study redox regulation of protein thiols. Plant Growth Regul..

[60] Dietz K.J. (2011). Peroxiredoxins in plants and cyanobacteria. Antioxid. Redox Signal..

[61] Foyer C.H., Noctor G. (2005). Redox homeostasis and antioxidant signaling: A metabolic interface between stress perception and physiological responses. Plant Cell.

[62] Haslekås C., Viken M.K., Grini P.E., Nygaard V., Nordgard S.H., Meza T.J., Aalen R.B. (2003). Seed 1-Cysteine peroxiredoxin antioxidants are not involved in dormancy, but contribute to inhibition of germination during stress. Plant Physiol..

[63] Dietz K.-J. (2003). Plant Peroxiredoxins. Annu. Rev. Plant Biol..

[64] Stacy R.A., Munthe E., Steinum T., Sharma B., Aalen R.B. (1996). A peroxiredoxin antioxidant is encoded by a dormancy-related gene, Per1, expressed during late development in the aleurone and embryo of barley grains. Plant Mol. Biol..

[65] Baier M., Dietz K.J. (1997). The plant 2-Cys peroxiredoxin BAS1 is a nuclear-encoded chloroplast protein: Its expressional regulation, phylogenetic origin, and implications for its specific physiological function in plants. Plant J..

[66] Goldmark P.J., Curry J., Morris C.F., Walker-Simmons M.K. (1992). Cloning and expression of an embryo-specific mRNA up-regulated in hydrated dormant seeds. Plant Mol. Biol..

[67] Aalen R.B., Opsahl-Ferstad H.G., Linnestad C., Olsen O.A. (1994). Transcripts encoding an oleosin and a dormancy-related protein are present in both the aleurone layer and the embryo of developing barley (*Hordeum vulgare* L.) seeds. Plant J..

[68] Haslekås C., Stacy R.A., Nygaard V., Culiáñez-Macià F.A., Aalen R.B. (1998). The expression of a peroxiredoxin antioxidant gene, *AtPer1*, in *Arabidopsis thaliana* is seed-specific and related to dormancy. Plant Mol. Biol..

[69] Lewis M.L., Miki K., Ueda T. (2000). *FePer 1*, a gene encoding an evolutionarily conserved 1-Cys peroxiredoxin in buckwheat (*Fagopyrum esculentum* Moench), is expressed in a seed-specific manner and induced during seed germination. Gene.

[70] Chen H., Chu P., Zhou Y., Ding Y., Li Y., Liu J., Jiang L., Huang S. (2016). Ectopic expression of NnPER1, a *Nelumbo nucifera* 1-cysteine peroxiredoxin antioxidant, enhances seed longevity and stress tolerance in Arabidopsis. Plant J..

[71] Serrato A.J., Cejudo F.J. (2003). Type-h thioredoxins accumulate in the nucleus of developing wheat seed tissues suffering oxidative stress. Planta.

[72] Kobrehel K., Wong J.H., Balogh A., Kiss F., Yee B.C., Buchanan B.B. (1992). Specific reduction of wheat storage proteins by thioredoxin h. Plant Physiol..

[73] Wong J.H., Kobrehel K., Buchanan B.B. (1995). Thioredoxin and seed proteins. Meth. Enzymol..

[74] Babazadeh N., Poursaadat M., Sadeghipour H.R., Hossein Zadeh Colagar A. (2012). Oil body mobilization in sunflower seedlings is potentially regulated by thioredoxin h. Plant Physiol. Biochem..

[75] Pulido P., Cazalis R., Cejudo F.J. (2009). An antioxidant redox system in the nucleus of wheat seed cells suffering oxidative stress. Plant J..

[76] Bernards M.A. (2002). Demystifying suberin. Can. J. Bot..

[77] Molina I., Ohlrogge J.B., Pollard M. (2008). Deposition and localization of lipid polyester in developing seeds of *Brassica napus* and *Arabidopsis thaliana*. Plant J..

[78] Daws M.I., Garwood N.C., Pritchard H.W. (2005). Traits of recalcitrant seeds in a semi-deciduous tropical forest in Panamá: Some ecological implications. Funct. Ecol..

[79] Pammenter N.W., Berjak P. (2000). Evolutionary and ecological aspects of recalcitrant seed biology. Seed Sci. Res..

[80] Tompsett P.B., Kemp R., Royal B.G. (1996). Database of Tropical Tree Seed Research, with Special Reference to the Dipterocarpaceae, Meliaceae and Araucariaceae: Database Contents.

[81] Debeaujon I., Léon-Kloosterziel K.M., Koornneef M. (2000). Influence of the Testa on Seed Dormancy, Germination, and Longevity in *Arabidopsis*. Plant Physiol..

[82] Lai Z., Vinod K., Zheng Z., Fan B., Chen Z. (2008). Roles of Arabidopsis WRKY3 and WRKY4 Transcription Factors in Plant Responses to Pathogens. BMC Plant Biol..

[83] Guo R., Qiao H., Zhao J., Wang X., Tu M., Guo C., Wan R., Li Z., Wang X. (2018). The Grape VlWRKY3 Gene Promotes Abiotic and Biotic Stress Tolerance in Transgenic *Arabidopsis thaliana*. Front. Plant Sci..

[84] Asano T., Masuda D., Yasuda M., Nakashita H., Kudo T., Kimura M., Yamaguchi K., Nishiuchi T. (2008). *AtNFXL1*, an *Arabidopsis* homologue of the human transcription factor NF-X1, functions as a negative regulator of the trichothecene phytotoxin-induced defense response. Plant J..

[85] Lisso J., Altmann T., Müssig C. (2006). The *AtNFXL1* gene encodes a NF-X1 type zinc finger protein required for growth under salt stress. FEBS Lett..

[86] Brauer E.K., Balcerzak M., Rocheleau H., Leung W., Schernthaner J., Subramaniam R., Ouellet T. (2019). Genome Editing of a Deoxynivalenol-Induced Transcription Factor Confers Resistance to *Fusarium graminearum* in Wheat. MPMI.

[87] Pourcel L., Routaboul J.-M., Kerhoas L., Caboche M., Lepiniec L., Debeaujon I. (2005). *TRANSPARENT TESTA10* Encodes a Laccase-Like Enzyme Involved in Oxidative Polymerization of Flavonoids in *Arabidopsis* Seed Coat. Plant Cell.

[88] Pourcel L., Routaboul J.-M., Cheynier V., Lepiniec L., Debeaujon I. (2007). Flavonoid oxidation in plants: From biochemical properties to physiological functions. Trends Plant Sci..

[89] Debeaujon I., Lepiniec L., Pourcel L., Routaboul J.-M., Bradford K.J., Nonogaki H. (2007). Seed Coat Development and Dormancy. Seed Development, Dormancy and Germination.

[90] Diederichsen A., Jones-Flory L.L. (2005). Accelerated aging tests with seeds of 11 flax (*Linum usitatissimum*) cultivars. Seed Sci. Technol..

[91] Zhang X.K., Yang G.T., Chen L., Yin J.M., Tang Z.L., Li J.N. (2006). Physiological differences between yellow-seeded and black-seeded rapeseed (*Brassica napus* L.) with different testa characteristics during artificial ageing. Seed Sci. Technol..

[92] Liang M., Davis E., Gardner D., Cai X., Wu Y. (2006). Involvement of *AtLAC15* in lignin synthesis in seeds and in root elongation of Arabidopsis. Planta.

[93] Tobimatsu Y., Chen F., Nakashima J., Escamilla-Treviño L.L., Jackson L., Dixon R.A., Ralph J. (2013). Coexistence but Independent Biosynthesis of Catechyl and Guaiacyl/Syringyl Lignin Polymers in Seed Coats[W][OPEN]. Plant Cell.

[94] Capeleti I., Ferrarese M.L.L., Krzyzanowski F.C., Ferrarese-Filho O. A New Procedure for Quantification of Lignin in Soybean (*Glycine max* (L.) Merrill) Seed Coat and Their Relationship with the Resistance to Mechanical Damage. https://www.ingentaconnect.com/content/ista/sst/2005/00000033/00000002/art00025.

[95] Zhang K., Lu K., Qu C., Liang Y., Wang R., Chai Y., Li J. (2013). Gene silencing of *bntt10* family genes causes retarded pigmentation and lignin reduction in the seed soat of *Brassica napus*. PLoS ONE.

[96] Pollard M., Beisson F., Li Y., Ohlrogge J.B. (2008). Building lipid barriers: Biosynthesis of cutin and suberin. Trends Plant Sci..

[97] Li-Beisson Y., Shorrosh B., Beisson F., Andersson M.X., Arondel V., Bates P.D., Baud S., Bird D., DeBono A., Durrett T.P. (2013). Acyl-Lipid Metabolism. ARBO J..

[98] Vishwanath S.J., Delude C., Domergue F., Rowland O. (2015). Suberin: Biosynthesis, regulation, and polymer assembly of a protective extracellular barrier. Plant Cell Rep..

[99] Beisson F., Li Y., Bonaventure G., Pollard M., Ohlrogge J.B. (2007). The acyltransferase GPAT5 is required for the synthesis of suberin in seed coat and root of *Arabidopsis*. Plant Cell.

[100] Bueso E., Muñoz-Bertomeu J., Campos F., Brunaud V., Martínez L., Sayas E., Ballester P., Yenush L., Serrano R. (2014). *Arabidopsis thaliana* HOMEOBOX25 Uncovers a role for gibberellins in seed longevity1[C][W]. Plant Physiol..

[101] Kosma D.K., Murmu J., Razeq F.M., Santos P., Bourgault R., Molina I., Rowland O. (2014). AtMYB41 activates ectopic suberin synthesis and assembly in multiple plant species and cell types. Plant J..

[102] Hu J., Mitchum M.G., Barnaby N., Ayele B.T., Ogawa M., Nam E., Lai W.-C., Hanada A., Alonso J.M., Ecker J.R. (2008). Potential sites of bioactive gibberellin production during reproductive growth in *Arabidopsis*. Plant Cell.

[103] Kim Y.-C., Nakajima M., Nakayama A., Yamaguchi I. (2005). Contribution of gibberellins to the formation of *Arabidopsis* seed coat through starch degradation. Plant Cell Physiol..

[104] Park D.H., Lim P.O., Kim J.S., Cho D.S., Hong S.H., Nam H.G. (2003). The *Arabidopsis COG1* gene encodes a Dof domain transcription factor and negatively regulates phytochrome signaling. Plant J..

[105] Hedden P., Thomas S.G. (2012). Gibberellin biosynthesis and its regulation. Biochem J..

[106] Battaglia M., Olvera-Carrillo Y., Garciarrubio A., Campos F., Covarrubias A.A. (2008). The enigmatic LEA proteins and other hydrophilins. Plant Physiol..

[107] Hong-Bo S., Zong-Suo L., Ming-An S. (2005). LEA proteins in higher plants: Structure, function, gene expression and regulation. Colloids Surf. B Biointerfaces.

[108] Battaglia M., Covarrubias A.A. (2013). Late Embryogenesis Abundant (LEA) proteins in legumes. Front. Plant Sci..

[109] Dure L., Greenway S.C., Galau G.A. Developmental Biochemistry of Cottonseed Embryogenesis and Germination: Changing Messenger Ribonucleic Acid Populations as Shown by In Vitro and In Vivo Protein Synthesis. https://pubs.acs.org/doi/pdf/10.1021/bi00517a033.

[110] Costa M.-C.D., Cooper K., Hilhorst H.W.M., Farrant J.M. (2017). Orthodox seeds and resurrection plants: Two of a Kind?. Plant Physiol..

[111] Li D., He X. (2009). Desiccation induced structural alterations in a 66-Amino Acid fragment of an anhydrobiotic Nematode Late Embryogenesis Abundant (LEA) Protein. Biomacromolecules.

[112] Tyson T., O’Mahony Zamora G., Wong S., Skelton M., Daly B., Jones J.T., Mulvihill E.D., Elsworth B., Phillips M., Blaxter M. (2012). A molecular analysis of desiccation tolerance mechanisms in the anhydrobiotic nematode *Panagrolaimus superbus* using expressed sequenced tags. BMC Res. Notes.

[113] LeBlanc B.M., Le M.T., Janis B., Menze M.A., Hand S.C. (2019). Structural properties and cellular expression of AfrLEA6, a group 6 late embryogenesis abundant protein from embryos of *Artemia franciscana*. Cell Stress Chaperones.

[114] Denekamp N.Y., Thorne M.A., Clark M.S., Kube M., Reinhardt R., Lubzens E. (2009). Discovering genes associated with dormancy in the monogonont rotifer *Brachionus plicatilis*. BMC Genom..

[115] Tripathi R., Boschetti C., McGee B., Tunnacliffe A. (2012). Trafficking of bdelloid rotifer late embryogenesis abundant proteins. J. Exp. Biol..

[116] Pedrosa A.M., de P.S. Martins C., Gonçalves L.P., Costa M.G.C. (2015). Late Embryogenesis Abundant (LEA) constitutes a large and diverse family of proteins involved in development and abiotic stress responses in sweet orange (*Citrus sinensis* L. Osb.). PLoS ONE.

[117] Gu W., Zhang A., Sun H., Gu Y., Chao J., Tian R., Duan J.-A. (2019). Identifying resurrection genes through the differentially expressed genes between *Selaginella tamariscina* (Beauv.) spring and *Selaginella moellendorffii* Hieron under drought stress. PLoS ONE.

[118] Zheng J., Su H., Lin R., Zhang H., Xia K., Jian S., Zhang M. (2019). Isolation and characterization of an atypical LEA gene (*IpLEA*) from *Ipomoea pes-caprae* conferring salt/drought and oxidative stress tolerance. Sci. Rep..

[119] Liu H., Xing M., Yang W., Mu X., Wang X., Lu F., Wang Y., Zhang L. (2019). Genome-wide identification of and functional insights into the late embryogenesis abundant (LEA) gene family in bread wheat (*Triticum aestivum*). Sci. Rep..

[120] Luo D., Hou X., Zhang Y., Meng Y., Zhang H., Liu S., Wang X., Chen R. (2019). CaDHN5, a Dehydrin Gene from Pepper, Plays an Important Role in Salt and Osmotic Stress Responses. Int. J. Mol. Sci.

[121] Olvera-Carrillo Y., Campos F., Reyes J.L., Garciarrubio A., Covarrubias A.A. (2010). functional analysis of the group 4 Late Embryogenesis Abundant proteins reveals their relevance in the adaptive response during water deficit in *Arabidopsis*. Plant Physiol..

[122] Farrant J.M., Pammenter N.W., Berjak P. (1993). Seed development in relation to desiccation tolerance: A comparison between desiccation-sensitive (recalcitrant) seeds of *Avicennia marina* and desiccation-tolerant types. Seed Sci. Res..

[123] Xu D., Duan X., Wang B., Hong B., Ho T.H.D., Wu R. (1996). Expression of a Late Embryogenesis Abundant protein gene, *HVA1*, from barley confers tolerance to water deficit and salt stress in transgenic rice. Plant Physiol..

[124] Iturriaga G. (2008). The LEA proteins and trehalose loving couple: A step forward in anhydrobiotic engineering. Biochem. J..

[125] Goyal K., Walton L.J., Tunnacliffe A. (2005). LEA proteins prevent protein aggregation due to water stress. Biochem. J..

[126] Furuki T., Sakurai M., Iwaya-Inoue M., Sakurai M., Uemura M. (2018). Physicochemical aspects of the biological functions of trehalose and group 3 LEA proteins as desiccation protectants. Survival Strategies in Extreme Cold and Desiccation: Adaptation Mechanisms and Their Applications.

[127] Hand S.C., Menze M.A., Toner M., Boswell L., Moore D. (2011). LEA proteins during water stress: Not Just for Plants Anymore. Annu. Rev. Physiol..

[128] Li H.-W., Zang B.-S., Deng X.-W., Wang X.-P. (2011). Overexpression of the trehalose-6-phosphate synthase gene *OsTPS1* enhances abiotic stress tolerance in rice. Planta.

[129] Dussert S., Serret J., Bastos-Siqueira A., Morcillo F., Déchamp E., Rofidal V., Lashermes P., Etienne H., JOët T. (2018). Integrative analysis of the late maturation programme and desiccation tolerance mechanisms in intermediate coffee seeds. J. Exp. Bot..

[130] Manfre A.J., LaHatte G.A., Climer C.R., Marcotte W.R. (2009). Seed dehydration and the establishment of desiccation tolerance during seed maturation is altered in the *Arabidopsis thaliana* mutant *atem6-1*. Plant Cell Physiol..

[131] Jin X., Liu D., Ma L., Gong Z., Cao D., Liu Y., Li Y., Jiang C. (2018). Transcriptome and expression profiling analysis of recalcitrant tea (*Camellia sinensis* L.) seeds sensitive to dehydration. Int. J. Genom..

[132] Covarrubias A.A., Romero-Pérez P.S., Cuevas-Velazquez C.L., Rendón-Luna D.F. (2020). The functional diversity of structural disorder in plant proteins. Arch. Biochem. Biophys..

[133] Zhu W., Zhang D., Lu X., Zhang L., Yu Z., Lv H., Zhang H. (2014). Characterisation of an SKn-type dehydrin promoter from wheat and its responsiveness to various abiotic and biotic Stresses. Plant Mol. Biol. Rep..

[134] Yoshida T., Mogami J., Yamaguchi-Shinozaki K. (2014). ABA-dependent and ABA-independent signaling in response to osmotic stress in plants. Curr. Opin. Plant Biol..

[135] Liu S., Lv Z., Liu Y., Li L., Zhang L., Liu S., Lv Z., Liu Y., Li L., Zhang L. (2018). Network analysis of ABA-dependent and ABA-independent drought responsive genes in *Arabidopsis thaliana*. Genet. Mol. Biol..

[136] Wang Z., Zhu Y., Wang L., Liu X., Liu Y., Phillips J., Deng X. (2009). A WRKY transcription factor participates in dehydration tolerance in *Boea hygrometrica* by binding to the W-box elements of the galactinol synthase (BhGolS1) promoter. Planta.

[137] Villalobos M.A., Bartels D., Iturriaga G. (2004). Stress tolerance and glucose insensitive phenotypes in *Arabidopsis* overexpressing the *CpMYB10* transcription factor gene. Plant Physiol..

[138] Suzuki M., Ketterling M.G., Li Q.-B., McCarty D.R. (2003). Viviparous1 alters global gene expression patterns through regulation of abscisic acid signaling. Plant Physiol..

[139] Finkelstein R.R., Gampala S.S.L., Rock C.D. (2002). Abscisic acid signaling in seeds and seedlings. Plant Cell.

[140] Xu P., Cai W. (2019). Function of *Brassica napus* BnABI3 in *Arabidopsis gs1*, an allele of AtABI3, in seed development and stress response. Front. Plant Sci..

[141] Rohde A., Kurup S., Holdsworth M. (2000). ABI3 emerges from the seed. Trends Plant Sci..

[142] Nakashima K., Yamaguchi-Shinozaki K. (2013). ABA signaling in stress-response and seed development. Plant Cell Rep..

[143] Carbonero P., Iglesias-Fernández R., Vicente-Carbajosa J. (2017). The AFL subfamily of B3 transcription factors: Evolution and function in angiosperm seeds. J. Exp. Bot..

[144] Suzuki M., McCarty D.R. (2008). Functional symmetry of the B3 network controlling seed development. Curr. Opin. Plant Biol..

[145] Vicente-Carbajosa J., Carbonero P. (2004). Seed maturation: Developing an intrusive phase to accomplish a quiescent state. Int. J. Dev. Biol..

[146] Fatihi A., Boulard C., Bouyer D., Baud S., Dubreucq B., Lepiniec L. (2016). Deciphering and modifying LAFL transcriptional regulatory network in seed for improving yield and quality of storage compounds. Plant Sci..

[147] Yamasaki K., Kigawa T., Seki M., Shinozaki K., Yokoyama S. (2013). DNA-binding domains of plant-specific transcription factors: Structure, function, and evolution. Trends Plant Sci..

[148] Braybrook S.A., Stone S.L., Park S., Bui A.Q., Le B.H., Fischer R.L., Goldberg R.B., Harada J.J. (2006). Genes directly regulated by LEAFY COTYLEDON2 provide insight into the control of embryo maturation and somatic embryogenesis. Proc. Natl. Acad. Sci. USA.

[149] Kroj T., Savino G., Valon C., Giraudat J., Parcy F. (2003). Regulation of storage protein gene expression in *Arabidopsis*. Development.

[150] Reidt W., Wohlfarth T., Ellerström M., Czihal A., Tewes A., Ezcurra I., Rask L., Bäumlein H. (2000). Gene regulation during late embryogenesis: The RY motif of maturation-specific gene promoters is a direct target of the FUS3 gene product. Plant J..

[151] Mönke G., Altschmied L., Tewes A., Reidt W., Mock H.-P., Bäumlein H., Conrad U. (2004). Seed-specific transcription factors ABI3 and FUS3: Molecular interaction with DNA. Planta.

[152] Ezcurra I., Wycliffe P., Nehlin L., Ellerström M., Rask L. (2000). Transactivation of the *Brassica napus* napin promoter by ABI3 requires interaction of the conserved B2 and B3 domains of ABI3 with different cis-elements: B2 mediates activation through an ABRE, whereas B3 interacts with an RY/G-box. Plant J..

[153] Grimault A., Gendrot G., Chaignon S., Gilard F., Tcherkez G., Thévenin J., Dubreucq B., Depège-Fargeix N., Rogowsky P.M. (2015). Role of B3 domain transcription factors of the AFL family in maize kernel filling. Plant Sci..

[154] Meinke D.W., Franzmann L.H., Nickle T.C., Yeung E.C. (1994). LEAFY COTYLEDON mutants of *Arabidopsis*. Plant Cell.

[155] Keith K., Kraml M., Dengler N.G., McCourt P. (1994). fusca3: A Heterochronic mutation affecting late embryo development in *Arabidopsis*. Plant Cell.

[156] Nambara E., Nambara E., McCourt P., Naito S. (1995). A regulatory role for the ABI3 gene in the establishment of embryo maturation in *Arabidopsis thaliana*. Development.

[157] Jo L., Pelletier J.M., Harada J.J. (2019). Central role of the LEAFY COTYLEDON1 transcription factor in seed development. J. Integr. Plant Biol..

[158] To A., Valon C., Savino G., Guilleminot J., Devic M., Giraudat J., Parcy F. (2006). A network of local and redundant gene regulation governs *Arabidopsis* seed maturation. Plant Cell.

[159] Kotak S., Vierling E., Bäumlein H., von Koskull-Döring P. (2007). A novel transcriptional cascade regulating expression of heat stress proteins during seed development of *Arabidopsis*. Plant Cell.

[160] Panchuk I.I., Volkov R.A., Schöffl F. (2002). Heat stress and heat shock transcription factor-dependent expression and activity of ascorbate peroxidase in *Arabidopsis*. Plant Physiol..

[161] Schramm F., Ganguli A., Kiehlmann E., Englich G., Walch D., von Koskull-Döring P. (2006). The heat stress transcription factor HsfA2 serves as a regulatory amplifier of a subset of genes in the heat stress response in *Arabidopsis*. Plant Mol. Biol..

[162] Li C., Chen Q., Gao X., Qi B., Chen N., Xu S., Chen J., Wang X. (2005). AtHsfA2 modulates expression of stress responsive genes and enhances tolerance to heat and oxidative stress in *Arabidopsis*. Sci. China Ser. C Life Sci..

[163] Panikulangara T.J., Eggers-Schumacher G., Wunderlich M., Stransky H., Schöffl F. (2004). Galactinol synthase 1. A novel heat shock factor target gene responsible for heat-induced synthesis of raffinose family oligosaccharides in *Arabidopsis*. Plant Physiol..

[164] Busch W., Wunderlich M., Schöffl F. (2005). Identification of novel heat shock factor-dependent genes and biochemical pathways in Arabidopsis thaliana. Plant J..

[165] Prieto-Dapena P., Castaño R., Almoguera C., Jordano J. (2006). Improved resistance to controlled deterioration in transgenic seeds. Plant Physiol..

[166] Verdier J., Lalanne D., Pelletier S., Torres-Jerez I., Righetti K., Bandyopadhyay K., Leprince O., Chatelain E., Vu B.L., Gouzy J. (2013). A regulatory network-based approach dissects late maturation processes related to the acquisition of desiccation tolerance and longevity of *Medicago truncatula* seeds. Plant Physiol..

[167] Mao Z., Sun W. (2015). *Arabidopsis* seed-specific vacuolar aquaporins are involved in maintaining seed longevity under the control of ABSCISIC ACID INSENSITIVE 3. J. Exp. Bot..

[168] Bies-Ethève N., Gaubier-Comella P., Debures A., Lasserre E., Jobet E., Raynal M., Cooke R., Delseny M. (2008). Inventory, evolution and expression profiling diversity of the LEA (late embryogenesis abundant) protein gene family in *Arabidopsis thaliana*. Plant Mol. Biol..

[169] Gubler F., Millar A.A., Jacobsen J.V. (2005). Dormancy release, ABA and pre-harvest sprouting. Curr. Opin. Plant Biol..

[170] Baud S., Mendoza M.S., To A., Harscoët E., Lepiniec L., Dubreucq B. (2007). WRINKLED1 specifies the regulatory action of LEAFY COTYLEDON2 towards fatty acid metabolism during seed maturation in *Arabidopsis*. Plant J..

[171] Che N., Yang Y., Li Y., Wang L., Huang P., Gao Y., An C. (2009). Efficient LEC2 activation of OLEOSIN expression requires two neighboring RY elements on its promoter. Sci. China Ser. C.

[172] Bensmihen S., Rippa S., Lambert G., Jublot D., Pautot V., Granier F., Giraudat J., Parcy F. (2002). The Homologous ABI5 and EEL transcription factors function antagonistically to fine-tune gene expression during late embryogenesis. Plant Cell.

[173] Wang F., Perry S.E. (2013). Identification of direct targets of fusca3, a key regulator of *Arabidopsis* seed development. Plant Physiol..

[174] Chen M., Zhang B., Li C., Kulaveerasingam H., Chew F.T., Yu H. (2015). TRANSPARENT TESTA GLABRA1 regulates the accumulation of seed storage reserves in *Arabidopsis*. Plant Physiol..

[175] Baud S., Dubreucq B., Miquel M., Rochat C., Lepiniec L. (2008). Storage reserve accumulation in *Arabidopsis*: Metabolic and developmental control of seed filling. Arab. Book.

[176] Yamamoto A., Kagaya Y., Usui H., Hobo T., Takeda S., Hattori T. (2010). Diverse roles and mechanisms of gene regulation by the *Arabidopsis* seed maturation master regulator FUS3 revealed by microarray analysis. Plant Cell Physiol..

[177] Tsai A.Y.-L., Gazzarrini S. (2012). AKIN10 and FUSCA3 interact to control lateral organ development and phase transitions in *Arabidopsis*. Plant J..

[178] Gutierrez L., Wuytswinkel O.V., Castelain M., Bellini C. (2007). Combined networks regulating seed maturation. Trends Plant Sci..

[179] Yamaguchi S., Kamiya Y., Nambara E. (2007). regulation of ABA and GA levels during seed development and germination in *Arabidopsis*. Annual Plant Reviews Volume 27: Seed Development, Dormancy and Germination.

[180] Braybrook S.A., Harada J.J. (2008). LECs go crazy in embryo development. Trends Plant Sci..

[181] Christmann A., Moes D., Himmelbach A., Yang Y., Tang Y., Grill E. (2006). Integration of abscisic acid signalling into plant responses. Plant Biol..

[182] Pawłowski T.A. (2009). Proteome analysis of Norway maple (*Acer platanoides* L.) seeds dormancy breaking and germination: Influence of abscisic and gibberellic acids. BMC Plant Biol..

[183] Hays D., Mandel R., Pharis R. (2001). Hormones in zygotic and microspore embryos of *Brassica napus*. Plant Growth Regul..

[184] Kagaya Y., Okuda R., Ban A., Toyoshima R., Tsutsumida K., Usui H., Yamamoto A., Hattori T. (2005). Indirect ABA-dependent regulation of seed storage protein genes by FUSCA3 transcription factor in *Arabidopsis*. Plant Cell Physiol..

[185] Gazzarrini S., Tsuchiya Y., Lumba S., Okamoto M., McCourt P. (2004). The transcription factor FUSCA3 controls developmental timing in *Arabidopsis* through the hormones gibberellin and abscisic acid. Dev. Cell.

[186] Ogawa M., Hanada A., Yamauchi Y., Kuwahara A., Kamiya Y., Yamaguchi S. (2003). Gibberellin biosynthesis and response during *Arabidopsis* seed germination. Plant Cell.

[187] Curaba J., Moritz T., Blervaque R., Parcy F., Raz V., Herzog M., Vachon G. (2004). *AtGA3ox2*, a key gene responsible for bioactive gibberellin biosynthesis, is regulated during embryogenesis by LEAFY COTYLEDON2 and FUSCA3 in *Arabidopsis*. Plant Physiol..

[188] Casson S.A., Lindsey K. (2006). The turnip mutant of *Arabidopsis* reveals that LEAFY COTYLEDON1 expression mediates the effects of auxin and sugars to promote embryonic cell identity. Plant Physiol..

[189] Ogas J., Cheng J.-C., Sung Z.R., Somerville C. (1997). cellular differentiation regulated by gibberellin in the *Arabidopsis thaliana pickle* mutant. Science.

[190] Rider S.D., Henderson J.T., Jerome R.E., Edenberg H.J., Romero-Severson J., Ogas J. (2003). Coordinate Repression of Regulators of Embryonic Identity by PICKLE during germination in *Arabidopsis*. Plant J..

[191] Bao Y., Song W.-M., Pan J., Jiang C.-M., Srivastava R., Li B., Zhu L.-Y., Su H.-Y., Gao X.-S., Liu H. (2016). Overexpression of the *NDR1*/*HIN1*-Like Gene *NHL6* modifies seed germination in response to abscisic acid and abiotic stresses in *Arabidopsis*. PLoS ONE.

[192] Garcia M.E., Lynch T., Peeters J., Snowden C., Finkelstein R. (2008). A small plant-specific protein family of ABI five binding proteins (AFPs) regulates stress response in germinating *Arabidopsis* seeds and seedlings. Plant Mol. Biol..

[193] Liu Y., He J., Chen Z., Ren X., Hong X., Gong Z. (2010). ABA overly-sensitive 5 (*ABO5*), encoding a pentatricopeptide repeat protein required for *cis*-splicing of mitochondrial *nad2* intron 3, is involved in the abscisic acid response in *Arabidopsis*. Plant J..

[194] Lim C.W., Baek W., Han S.-W., Lee S.C. (2013). *Arabidopsis* PYL8 plays an important role for ABA signaling and drought stress responses. Plant Pathol. J..

